# Supplementary material for: Blood Biochemical Responses to Acute Exercise: Findings from the Molecular Transducers of Physical Activity Consortium (MoTrPAC)
Source: bioRxiv. 2026 Mar 11:2026.03.02.704798. Preprint. [Version 2] doi: 10.64898/2026.03.02.704798 (PMC12980391; doi:10.64898/2026.03.02.704798)
Supplement: Supplement 2 [file media-2.docx]

# Key Resources Table

| REAGENT or RESOURCE | SOURCE | IDENTIFIER |
| --- | --- | --- |
| Biological samples | | |
| Human skeletal muscle samples | This paper | MoTrPAC Human PreCOVID Dataset |
| Human adipose tissue samples | This paper | MoTrPAC Human PreCOVID Dataset |
| Human blood samples (PAXgene and plasma) | This paper | MoTrPAC Human PreCOVID Dataset |
| Chemicals, peptides, and recombinant proteins | | |
| 8M Urea | Sigma-Aldrich | Catalog: U4883-6X25ML |
| Tris pH 8.0 | Invitrogen | Catalog: AM9855G |
| Sodium chloride | Sigma-Aldrich | Catalog: 71376 |
| EDTA | Sigma-Aldrich | Catalog: E7889 |
| Aprotinin | Sigma-Aldrich | Catalog: A6103 |
| Leupeptin | Roche CustomBiotech | Catalog: 11017101001 |
| PMSF | Sigma-Aldrich | Catalog: 78830 |
| Sodium fluoride | Sigma-Aldrich | Catalog: S7920 |
| Phosphatase inhibitor cocktail 2 | Sigma-Aldrich | Catalog: P5726 |
| Phosphatase inhibitor cocktail 3 | Sigma-Aldrich | Catalog: P0044 |
| Dithiothreitol | Sigma-Aldrich | Catalog: 20291 |
| Iodoacetamide | Sigma-Aldrich | Catalog: A3221 |
| LysC endopeptidase | Wako Chemicals | Catalog: 129-02541 |
| Trypsin | Promega | Catalog: V511X |
| Formic acid | Sigma-Aldrich | Catalog: F0507 |
| Acetonitrile (LC-MS grade) | Honeywell | Catalog: 34967 |
| TMT 16-plex reagent | ThermoFisher | Catalog: A34808 |
| HEPES pH 8.5 | Alfa Aesar | Catalog: J63218 |
| Hydroxylamine solution, 50% | Aldrich | Catalog: 467804 |
| Methanol | Honeywell | Catalog: 34966 |
| Ammonium formate | Sigma-Aldrich | Catalog: 338818 |
| Sep-Pak C18 columns | Waters | Catalog: WAT054925 |
| Critical Commercial Assays | | |
| TruSeq Methyl Capture EPIC Library Prep Kit | Illumina | Catalog: FC-151-1003 |
| Universal Plus mRNA-Seq kit | NuGEN/Tecan | Catalog: 9133 |
| Agencourt RNAdvance tissue lysis buffer | Beckman Coulter | Catalog: A48706 |
| Agencourt RNAdvance blood specific kit | Beckman Coulter | Catalog: A48707 |
| GenFind v3 kit | Beckman Coulter | Catalog: A48708 |
| PAXgene tubes | BD Biosciences | Catalog: 762165 |
| BCA Protein Assay Kit | ThermoFisher | Catalog: 23225 |
| MassTrak kit | Waters | Catalog: 186007836 |
| Deposited Data | | |
| MoTrPAC Human PreCOVID Dataset | MoTrPAC DataHub | motrpac-data.org |
| Software and Algorithms | | |
| bcl2fastq2 v2.20.0 | Illumina | https://support.illumina.com/sequencing/sequencing_software/bcl2fastq-conversion-software.html |
| BWA-MEM v0.7.15 | Li, H.# | https://github.com/lh3/bwa |
| GATK v3.5 | McKenna et al.# | https://github.com/broadinstitute/gatk |
| bismark v0.20.0 | Krueger and Andrews.# | https://github.com/FelixKrueger/Bismark |
| NGSCheckmate v1.01 | Lee et al.# | https://github.com/parklab/NGSCheckMate |
| samtools | Danecek et al.# | https://www.htslib.org/ |
| Markov Cluster Algorithm (MCL) | Dongen, S.V.# | https://cran.r-project.org/web/packages/MCL/index.html |
| ENCODE ATAC-seq pipeline v1.7.0 | ENCODE.# | https://github.com/ENCODE-DCC/atac-seq-pipeline |
| cutadapt v1.18, v2.5 | Martin, M.# | https://cutadapt.readthedocs.io/ |
| Bowtie 2 v2.3.4.3 | Langmead & Salzberg.# | http://bowtie-bio.sourceforge.net/bowtie2/index.shtml |
| MACS2 v2.2.4 | Zhang et al.# | https://github.com/macs3-project/MACS |
| bedtools v2.29.0 | Quinlan & Hall.# | https://bedtools.readthedocs.io/ |
| STAR v2.7.0d | Dobin et al.# | https://github.com/alexdobin/STAR |
| RSEM v1.3.1 | Li & Dewey.# | https://github.com/deweylab/RSEM |
| FastQC v0.11.8 | Babraham Institute.# | https://www.bioinformatics.babraham.ac.uk/projects/fastqc/ |
| Picard v2.4.1, v2.18.16 | Broad Institute.# | https://github.com/broadinstitute/picard |
| MultiQC v1.6 | Seqera | https://multiqc.info/ |
| limma | Ritchie et al.# | https://bioconductor.org/packages/release/bioc/html/limma.html |
| edgeR | Robinson et al.# | https://bioconductor.org/packages/release/bioc/html/edgeR.html |
| variancePartition | Hoffman & Roussos.# | https://bioconductor.org/packages/release/bioc/html/variancePartition.html |
| ChIPseeker | Yu et al.# | https://bioconductor.org/packages/release/bioc/html/ChIPseeker.html |
| biomaRt v2.58.2 | Durinck et al.# | https://bioconductor.org/packages/release/bioc/html/biomaRt.html |
| Skyline v25.1.0.142 | Pino et al.# | https://skyline.ms/ |
| TraceFinder | ThermoFisher | https://www.thermofisher.com/us/en/home/industrial/mass-spectrometry/liquid-chromatography-mass-spectrometry-lc-ms/lc-ms-software/lc-ms-data-acquisition-software/tracefinder-software.html |
| Progenesis QI | Nonlinear Dynamics | https://www.nonlinear.com/progenesis/qi/ |
| CAMERA-PR | Wu & Smyth.# | https://bioconductor.org/packages/release/bioc/html/CAMERA.html |
| PLIER | Mao et al.# | https://github.com/wgmao/PLIER |
| HOMER v4.11 | Heinz et al.# | http://homer.ucsd.edu/homer/ |
| SC-ION | Clark et al.# | https://github.com/nmclark2/SCION |
| TMSig | Sagendorf, T.# | https://www.bioconductor.org/packages/release/bioc/html/TMSig.html |
| Mfuzz | Kumar and Futschik.# | https://bioconductor.org/packages/release/bioc/html/Mfuzz.html |
| KEGGREST | Tenenbaum and Maintainer.# | https://www.bioconductor.org/packages/release/bioc/html/KEGGREST.html |
| Metabolomics Workbench | UCSD | https://www.metabolomicsworkbench.org/ |
| PhosphositePlus | Hornbeck et al.# | https://www.phosphosite.org/ |
| MSigDB v2023.2.Hs | Liberzon et al.# | https://www.gsea-msigdb.org/gsea/msigdb |
| MitoCarta3.0 | Rath et al.# | https://www.broadinstitute.org/mitocarta/mitocarta30-inventory-mammalian-mitochondrial-proteins-and-pathways |
| CellMarker2.0 | Hu et al.# | http://bio-bigdata.hrbmu.edu.cn/CellMarker/ |
| MoTrPAC Methyl Capture Pipeline | MoTrPAC | https://github.com/MoTrPAC/motrpac-methyl-capture-pipeline |
| MoTrPAC Rat Training 6mo R Package | MoTrPAC Study Group.# | https://github.com/MoTrPAC/MotrpacRatTraining6mo/ |
| MoTrPAC Human PreSuspensionData R Package | MoTrPAC | https://github.com/MoTrPAC/MotrpacHumanPreSuspensionData |
| MoTrPAC Human PreSuspension R Package | MoTrPAC | https://github.com/MoTrPAC/MotrpacHumanPreSuspension |
| MoTrPAC PreCOVID Analyses Repository | GitHub | https://github.com/MoTrPAC/precovid-analyses |
| Google Cloud Platform | Google | https://cloud.google.com/ |
| Other | | |
| Illumina NovaSeq 6000 | Illumina | Catalog: 20012850 |
| Q Exactive Plus Mass Spectrometer | ThermoFisher | Catalog: IQLAAEGAAPFALGMBDK |
| Q Exactive HF-X Mass Spectrometer | ThermoFisher | Catalog: 0726042 |
| Orbitrap Fusion Lumos Tribrid Mass Spectrometer | ThermoFisher | Catalog: IQLAAEGAAPFADBMBHQ |
| Thermo Orbitrap Exploris 480 | ThermoFisher | Catalog: BRE725539 |
| Xevo TQ-S Mass Spectrometer | Waters | Catalog: 715001734 |
| Xevo TQ-XS Mass Spectrometer | Waters | Catalog: 715001735 |
| Quantum Ultra Mass Spectrometer | ThermoFisher | Catalog: IQLAAEGAA PFADBMBHQ |
| Agilent 1290 Infinity II | Agilent | Catalog: G4220A |
| Agilent 6495 Mass Spectrometer | Agilent | Catalog: G6495A |
| Agilent 6545 qTOF MS | Agilent | Catalog: G6545A |
| Agilent 5977B GC/MS | Agilent | Catalog: G5977B |
| Shimadzu Nexera X2 UHPLC | Shimadzu | Catalog: LC-30AD |
| Acquity UPLC | Waters | Catalog: ACQUITY-UPLC |
| Acquity HSS T3 column | Waters | Catalog: 186003539 |
| Atlantis HILIC column | Waters | Catalog: 186003539 |
| Zorbax Extend C18 column | Agilent | Catalog: 959700-902 |
| Chromolith FastGradient RP-18e column | EMD Millipore | Catalog: 1.52002.0001 |
| Accucore C18 column | ThermoFisher | Catalog: 17126-152130 |
| BiomekFX automation workstation | Beckman Coulter | Catalog: A48709 |
| Biomek i7 laboratory automation | Beckman Coulter | Catalog: A48710 |
| Covaris E220 ultrasonicator | Covaris | Catalog: 500217 |
| Bioanalyzer High Sensitivity DNA chip | Agilent | Catalog: 5067-4626 |
| Qubit dsDNA BR assay | ThermoFisher | Catalog: Q32850 |
| NanoDrop | ThermoFisher | Catalog: ND-ONE-W |
| Fragment Analyzer | Agilent | Catalog: M5310AA |
| TissueLyser II | QIAGEN | Catalog: 85300 |
| Omni bead ruptor | Omni International | Catalog: 19-040E |
| Branson 450 probe sonicator | Branson | Catalog: 101-063-066 |
| Biotage Extrahera | Biotage | Catalog: 400001 |
| Isolute C18 SPE columns | Biotage | Catalog: 400001 |
| Vacuum centrifuge | ThermoFisher | Catalog: SPD121P-115 |
| Centrifuge | Eppendorf | Catalog: 5427 R |
| Benchtop mini centrifuge | Corning | Catalog: 6765 |
| Benchtop vortex | Scientific Industries | Catalog: SI-0236 |
| Incubating shaker | VWR | Catalog: 12620-942 |
| Thermomixer | Eppendorf | Catalog: 5355 000.011 |
| Microplate Reader | Molecular Devices | Catalog: M2 |
| 96-well microplate | Greiner | Catalog: 655101 |
| Microplate foil cover | Corning | Catalog: PCR-AS-200 |
| 1.5 mL microtube w/o cap | Sarstedt | Catalog: 72.607 |
| 2.0 mL microtube w/o cap | Sarstedt | Catalog: 72.608 |
| Microtube caps | Sarstedt | Catalog: 72.692 |
| 1.5 mL snapcap tube | ThermoFisher | Catalog: AM12450 |
| 2.0 mL snapcap tube | ThermoFisher | Catalog: AM12475 |
| 15 mL centrifuge tube | Corning | Catalog: 352097 |
| 50 mL centrifuge tube | Corning | Catalog: 352070 |
| 300 µL LC-MS autosampler vial and cap | Waters | Catalog: 186002639 |
| 700 µL bRP fractionation autosampler vial | ThermoFisher | Catalog: C4010-14 |
| 700 µL bRP fractionation autosampler cap | ThermoFisher | Catalog: C4010-55A |
| Offline 96-well fractionation plate | Whatman | Catalog: 77015200 |
