# Supplementary material for: Blood Biochemical Responses to Acute Exercise: Findings from the Molecular Transducers of Physical Activity Consortium (MoTrPAC)
Source: bioRxiv. 2026 Mar 11:2026.03.02.704798. Preprint. [Version 2] doi: 10.64898/2026.03.02.704798 (PMC12980391; doi:10.64898/2026.03.02.704798)
Supplement: Supplement 3 [file NIHPP2026.03.02.704798v2-supplement-3.pdf]

bioRxiv preprint doi: https://doi.org/10.64898/2026.03.02.704798; this version posted March 11, 2026. The copyright holder for this preprint (which was not certified by peer review) is the author/funder, who has granted bioRxiv a license to display the preprint in perpetuity. It is made available under aCC-BY 4.0 International license.

Figure S1. Blood biochemical changes in response to acute endurance and resistance exercise according to blood biochemical profiling platform and in non-exercise controls.

(A) UpSet plots showing differentially abundant (DA) features for each blood or plasma biochemical profiling method across all EE and RE time points. Black dots indicate the plotted

bioRxiv preprint doi: https://doi.org/10.64898/2026.03.02.704798; this version posted March 11, 2026. The copyright holder for this preprint (which was not certified by peer review) is the author/funder, who has granted bioRxiv a license to display the preprint in perpetuity. It is made available under aCC-BY 4.0 International license.

group. A connected line between black dots indicates that the plotted bar represents features shared between those groups. The UpSet plot is ordered by the greatest number of features.

(B) Histogram showing molecular changes in the non-exercise controls at each time point in each ome (p-adj <0.05).

(C) Volcano plot showing all metabolite features changing in the control group at each time point. Features are plotted according to their log fold-change (X-axis) and -log10(p-value) Y-axis. Selected features are labeled.

Figure S2. Metabolite class enrichment by exercise mode and time point

(A-C) Metabolite class enrichment analysis using CAMERA-PR applied to metabolites features at each mode-time point pair. Panels show enrichment results for (A) all annotated metabolite classes that display an exercise effect, (B) C24 bile acids, and (C) xanthines. The plotted bubbles indicate the z-score (color/intensity) and significance (grey/white background) of the enrichment term; an * denotes p-adj <0.05. The key for post-exercise timepoint sampling and modalities is presented in panel A.

bioRxiv preprint doi: https://doi.org/10.64898/2026.03.02.704798; this version posted March 11, 2026. The copyright holder for this preprint (which was not certified by peer review) is the author/funder, who has granted bioRxiv a license to display the preprint in perpetuity. It is made available under aCC-BY 4.0 International license.

Figure S3. Differential plasma metabolomic responses to EE and RE

(A) Metabolite changes at post 10 min between EE vs CON (log fold-change on X-axis) and RE vs CON (log fold-change on y-axis). (B) Exercise mode-divergent metabolites (circled metabolites in (A) that are FDR significant for EE and RE) highlighting plasma medium- and long-chain acylcarnitine and fatty acid responses (orange lines reflect EE response, green lines reflect RE responses).

bioRxiv preprint doi: https://doi.org/10.64898/2026.03.02.704798; this version posted March 11, 2026. The copyright holder for this preprint (which was not certified by peer review) is the author/funder, who has granted bioRxiv a license to display the preprint in perpetuity. It is made available under aCC-BY 4.0 International license.

Figure S5. Comparison of resistance versus endurance exercise on plasma proteins

(A) Distribution of baseline (resting) eGFR correlations for 95 proteins that are DA at 40 minutes during exercise in EE.

(B-D) Four-quadrant plots displaying log fold-change of DA proteins in EE (x-axis) and RE (y-axis) at (B) post 10 min, (C) post 30 min, and (D) post 3.5 h time points. Corresponding bar charts with DA features in each group (RE, green arrows; EE, orange arrows) that compare the magnitude of exercise effect between modes.

bioRxiv preprint doi: https://doi.org/10.64898/2026.03.02.704798; this version posted March 11, 2026. The copyright holder for this preprint (which was not certified by peer review) is the author/funder, who has granted bioRxiv a license to display the preprint in perpetuity. It is made available under aCC-BY 4.0 International license.

Figure S6. Tissue-sources of plasma proteins that change in late exercise recovery

(A-B) (A) Heatmap of all DA plasma proteins at the 3.5 h timepoint show plasma protein changes, and skeletal muscle and adipose mRNA and global proteomic changes in EE and RE in MoTrPAC: *p-adj <0.05. Light grey boxes reflect time points not sampled. Dark grey boxes reflect features not detected. Red arrow highlighting that TNFRSF8 (CD30), a protein annotated to adipose tissue, shows no change in adipose mRNA expression during exercise but (B) decreased skeletal muscle mRNA expression after RE and EE with a subsequent decrease and trend towards decrease in plasma protein levels after RE and EE, respectively. Points with black dots indicate significance (p-adj <0.05) by linear mixed effects model. Error bars indicate 95% confidence intervals.

bioRxiv preprint doi: https://doi.org/10.64898/2026.03.02.704798; this version posted March 11, 2026. The copyright holder for this preprint (which was not certified by peer review) is the author/funder, who has granted bioRxiv a license to display the preprint in perpetuity. It is made available under aCC-BY 4.0 International license.

Figure S7. Differences in blood transcriptional responses to acute endurance and resistance exercise.

(A) Log2 fold-change in transcriptional abundance from pre-exercise, relative to control are shown for EE (orange lines) and RE (green lines) over time in adipose, blood, and skeletal muscle. Points with black dots indicate significance (p-adj <0.05) by linear mixed effects model. Error bars indicate 95% confidence intervals.

(B) Heatmap depicting z-scores of differential abundance of features in the Reactome HSF1 activation pathway according to exercise mode and time point for blood; an * denotes significance at p-adj <0.05 by linear mixed effects model.

bioRxiv preprint doi: https://doi.org/10.64898/2026.03.02.704798; this version posted March 11, 2026. The copyright holder for this preprint (which was not certified by peer review) is the author/funder, who has granted bioRxiv a license to display the preprint in perpetuity. It is made available under aCC-BY 4.0 International license.

Figure S8. Feature level immune cell enrichments displaying mode-specific regulation.

(A-F) Heatmaps depicting z-scores of differential abundance of features in CellMarker 2.0 (A) eosinophil, (B) granulocyte, and (C) platelet human blood transcriptomic pathway enrichments according to exercise mode and time point for blood. Manually curated heatmaps representing transcriptomic markers of: (D) monocytes (E) NK cells, and (F) T cells. In (D), FCGR3A and FCGR3B represent CD16A and CD16B, respectively. Select MHC Class II genes are by select HLA subclasses, with CD11B denoted as ITGAM. In all figures, an * denotes significance at p-adj <0.05 by linear mixed effects model.

bioRxiv preprint doi: https://doi.org/10.64898/2026.03.02.704798; this version posted March 11, 2026. The copyright holder for this preprint (which was not certified by peer review) is the author/funder, who has granted bioRxiv a license to display the preprint in perpetuity. It is made available under aCC-BY 4.0 International license.

Table S1. Clinical Characteristics of the MoTrPAC Pre-COVID Blood Cohort by Biochemical Profiling Platform

	Transcriptomics				Proteomics				Metabolomics			
**Characteristic**	**Overall** N=173	**CON** N=37	**EE** N=64	**RE** N=72	**Overall** N=44	**CON** N=12	**EE** N=14	**RE** N=18	**Overall** N=175	**CON** N=37	**EE** N=65	**RE** N=73
Sex												
Female	125 (72%)	31 (84%)	46 (72%)	48 (67%)	33 (75%)	9 (75%)	11 (79%)	13 (72%)	126 (72%)	31 (84%)	46 (71%)	49 (67%)
Male	48 (28%)	6 (16%)	18 (28%)	24 (33%)	11 (25%)	3 (25%)	3 (21%)	5 (28%)	49 (28%)	6 (16%)	19 (29%)	24 (33%)
Age (yrs)	41 (15)	42 (15)	41 (14)	40 (15)	43 (16)	52 (13)	40 (18)	39 (15)	41 (15)	42 (15)	42 (14)	40 (15)
Height (cm)	168 (9)	167 (8)	168 (9)	169 (10)	166 (10)	166 (9)	166 (11)	166 (10)	168 (9)	167 (8)	168 (9)	168 (10)
Weight (kg)	76 (14)	73 (13)	76 (13)	77 (14)	73 (12)	71 (11)	75 (15)	73 (10)	76 (14)	73 (13)	76 (13)	77 (14)
BMI (kg/m²)	26.9 (4.0)	26.2 (3.9)	27.0 (4.0)	27.1 (4.0)	26.6 (3.5)	25.8 (3.2)	27.4 (4.1)	26.6 (3.2)	26.9 (4.0)	26.2 (3.9)	27.0 (4.0)	27.2 (4.0)
Waist Circumference (cm)	92 (12)	89 (13)	92 (11)	93 (12)	91 (10)	88 (11)	92 (12)	92 (8)	92 (12)	89 (13)	93 (11)	93 (12)
Systolic BP (mmHg)	116 (12)	114 (11)	117 (11)	116 (12)	117 (11)	119 (13)	120 (10)	113 (11)	116 (12)	114 (11)	117 (11)	116 (12)
Diastolic BP (mmHg)	72 (8)	72 (8)	73 (9)	72 (8)	71 (8)	73 (9)	74 (7)	69 (8)	72 (8)	72 (8)	73 (9)	72 (8)

bioRxiv preprint doi: https://doi.org/10.64898/2026.03.02.704798; this version posted March 11, 2026. The copyright holder for this preprint (which was not certified by peer review) is the author/funder, who has granted bioRxiv a license to display the preprint in perpetuity. It is made available under aCC-BY 4.0 International license.

Hemoglobin A1c (%)	5.29 (0.30)	5.29 (0.21)	5.28 (0.31)	5.31 (0.32)	5.33 (0.29)	5.33 (0.18)	5.31 (0.37)	5.35 (0.28)	5.30 (0.30)	5.29 (0.21)	5.29 (0.32)	5.31 (0.32)
eGFR (mL/min/1.73 m²)	101 (20)	99 (19)	103 (18)	101 (22)	101 (18)	94 (18)	107 (19)	100 (17)	101 (20)	99 (19)	103 (18)	101 (22)
**Cardiopulmonary Exercise Test (CPET)**												
VO2peak (L/min)	1.86 (0.65)	1.73 (0.56)	1.90 (0.69)	1.90 (0.66)	1.82 (0.73)	1.64 (0.53)	1.92 (0.79)	1.86 (0.80)	1.86 (0.65)	1.73 (0.56)	1.90 (0.68)	1.89 (0.65)
VO2peak (ml/min/kg)	25 (7)	24 (6)	25 (7)	25 (7)	25 (8)	23 (6)	25 (9)	25 (9)	24 (7)	24 (6)	25 (7)	25 (7)
Workload (Watts)	152 (51)	142 (40)	156 (54)	154 (53)	148 (59)	136 (42)	157 (63)	149 (68)	152 (50)	142 (40)	155 (53)	153 (52)
O2 Pulse (ml/beat)	10.7 (3.3)	10.0 (2.8)	10.9 (3.5)	10.8 (3.4)	10.4 (3.6)	9.9 (3.1)	10.8 (3.8)	10.5 (3.9)	10.6 (3.3)	10.0 (2.8)	10.9 (3.5)	10.8 (3.4)
RER	1.16 (0.08)	1.16 (0.08)	1.17 (0.07)	1.15 (0.08)	1.15 (0.07)	1.18 (0.09)	1.15 (0.05)	1.12 (0.07)	1.16 (0.08)	1.16 (0.08)	1.17 (0.07)	1.15 (0.08)
**Strength Tests**												
Isometric Knee Extension Torque (Nm)	147 (57)	137 (53)	143 (53)	156 (60)	145 (44)	148 (39)	145 (53)	141 (42)	148 (57)	137 (53)	145 (54)	156 (60)
Hand Grip Strength (kg)	30 (11)	29 (8)	30 (11)	31 (12)	28 (12)	29 (11)	28 (12)	27 (13)	30 (11)	29 (8)	30 (11)	31 (12)

[1] n (%); Mean (SD)

bioRxiv preprint doi: https://doi.org/10.64898/2026.03.02.704798; this version posted March 11, 2026. The copyright holder for this preprint (which was not certified by peer review) is the author/funder, who has granted bioRxiv a license to display the preprint in perpetuity. It is made available under aCC-BY 4.0 International license.

Table S2. Clinical Characteristics of the MoTrPAC Pre-COVID Blood Cohort by Sex

**Characteristic**	**Female**<br>N = 126[1]	**Male**<br>N = 49[1]
Group		
CON	31 (25%)	6 (12%)
EE	46 (37%)	19 (39%)
RE	49 (39%)	24 (49%)
Age (yrs)	41 (14)	42 (16)
Height (cm)	164 (6)	178 (7)
Weight (kg)	73 (12)	85 (13)
BMI (kg/m²)	26.9 (4.1)	26.9 (3.7)
Waist Circumference (cm)	90 (11)	96 (11)
Systolic BP (mmHg)	114 (11)	121 (11)
Diastolic BP (mmHg)	71 (8)	75 (9)
Hemoglobin A1c (%)	5.30 (0.31)	5.28 (0.28)
eGFR (mL/min/1.73 m²)	102 (19)	100 (21)
**Cardiopulmonary Exercise Test (CPET)**		
VO2peak (L/min)	1.58 (0.38)	2.59 (0.63)
VO2peak (ml/min/kg)	22 (5)	31 (7)
Workload (Watts)	130 (31)	208 (47)
O2 Pulse (ml/beat)	9.2 (1.9)	14.4 (3.2)
RER	1.15 (0.08)	1.17 (0.07)

bioRxiv preprint doi: https://doi.org/10.64898/2026.03.02.704798; this version posted March 11, 2026. The copyright holder for this preprint (which was not certified by peer review) is the author/funder, who has granted bioRxiv a license to display the preprint in perpetuity. It is made available under aCC-BY 4.0 International license.

**Strength Tests**		
Isometric Knee Extension Torque (Nm)	129 (43)	196 (58)
Hand Grip Strength (kg)	26 (7)	42 (10)

[1] n (%); Mean (SD)

Table S3. Metabolites by c-means clustering analyses

Table S4. Plasma protein associations with baseline estimated glomerular filtration rate

Figure S1

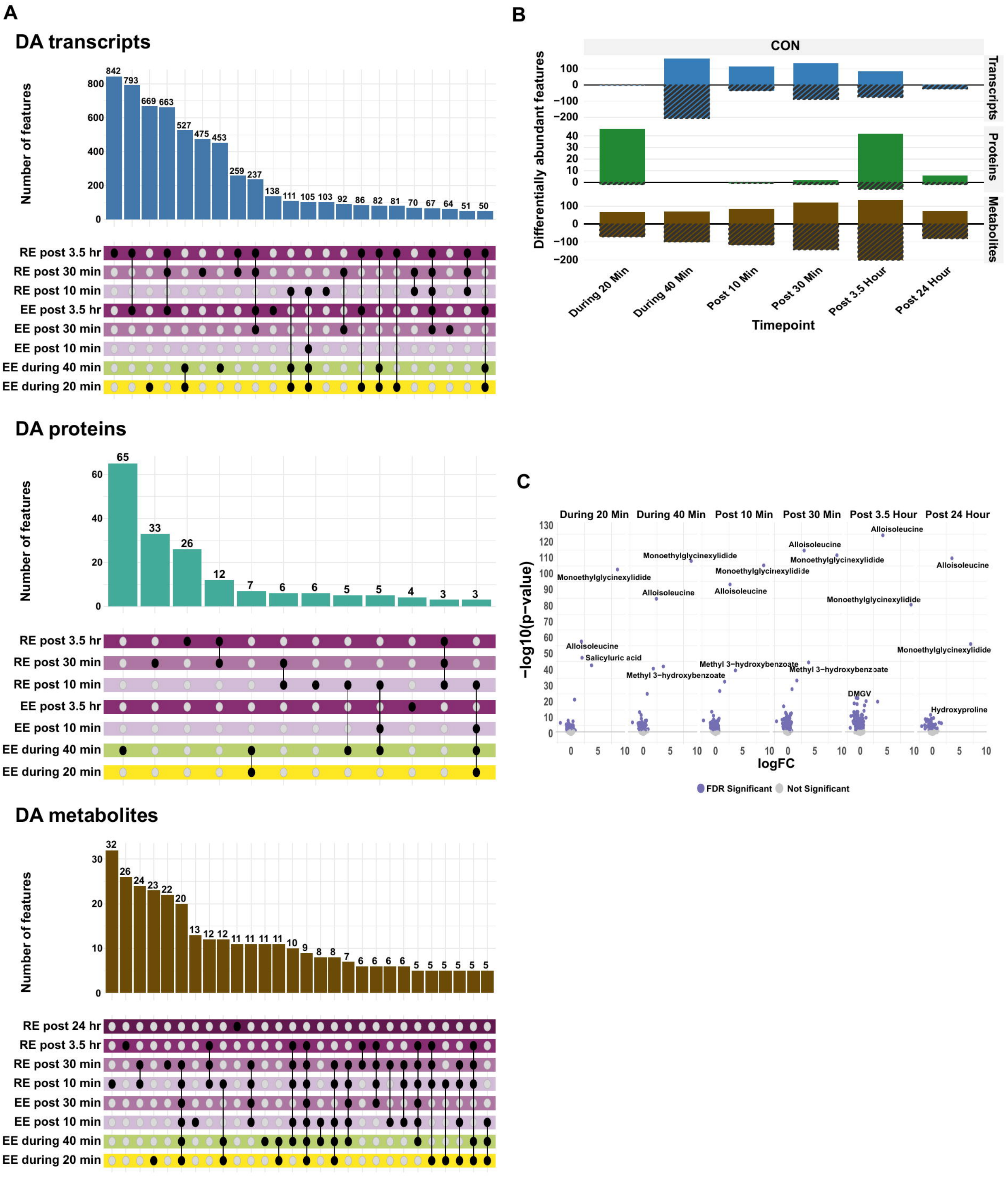
A
DA transcripts
Number of features
842
793
669
663
527
475
453
259
237
138
111
105
103
92
86
82
81
70
67
64
51
50
RE post 3.5 hr
RE post 30 min
RE post 10 min
EE post 3.5 hr
EE post 30 min
EE post 10 min
EE during 40 min
EE during 20 min
DA proteins
Number of features
65
33
26
12
7
6
6
5
5
4
3
3
RE post 3.5 hr
RE post 30 min
RE post 10 min
EE post 3.5 hr
EE post 10 min
EE during 40 min
EE during 20 min
DA metabolites
Number of features
32
26
24
23
22
20
13
12
12
11
11
11
11
10
9
8
8
7
6
6
6
6
5
5
5
5
5
5
RE post 24 hr
RE post 3.5 hr
RE post 30 min
RE post 10 min
EE post 30 min
EE post 10 min
EE during 40 min
EE during 20 min
B
CON
Differentially abundant features
Transcripts
Proteins
Metabolites
During 20 Min
During 40 Min
Post 10 Min
Post 30 Min
Post 3.5 Hour
Post 24 Hour
Timepoint
C
During 20 Min
During 40 Min
Post 10 Min
Post 30 Min
Post 3.5 Hour
Post 24 Hour
−log10(p−value)
Monoethylglycinexylidide
Alloisoleucine
Salicyluric acid
Methyl 3−hydroxybenzoate
DMGV
Hydroxyproline
logFC
FDR Significant
Not Significant

Figure S2

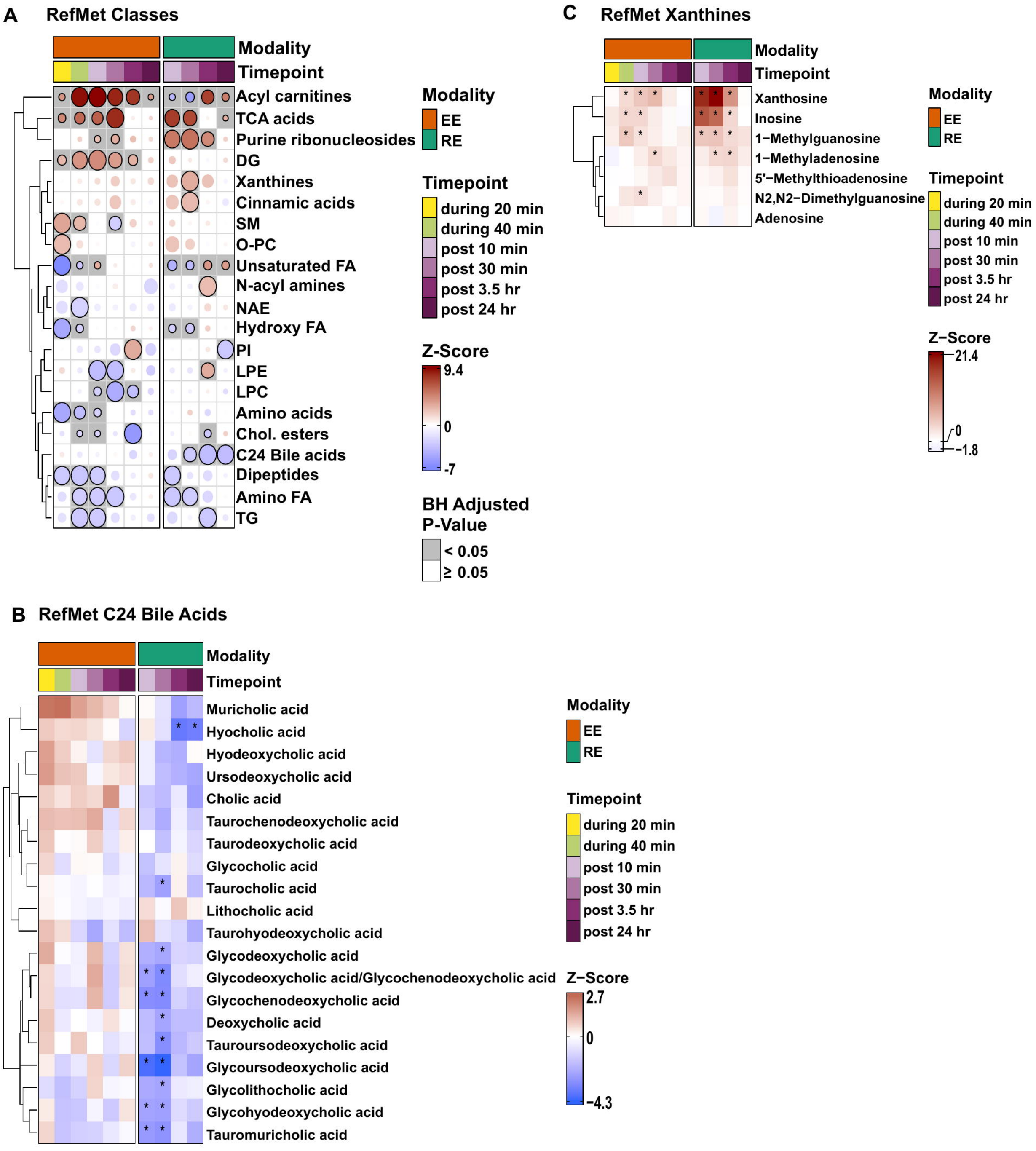

A RefMet Classes
Modality
Timepoint
Acyl carnitines
TCA acids
Purine ribonucleosides
DG
Xanthines
Cinnamic acids
SM
O-PC
Unsaturated FA
N-acyl amines
NAE
Hydroxy FA
PI
LPE
LPC
Amino acids
Chol. esters
C24 Bile acids
Dipeptides
Amino FA
TG
Modality
EE
RE
Timepoint
during 20 min
during 40 min
post 10 min
post 30 min
post 3.5 hr
post 24 hr
Z-Score
9.4
0
-7
BH Adjusted P-Value
< 0.05
≥ 0.05
C RefMet Xanthines
Modality
Timepoint
Xanthosine
Inosine
1−Methylguanosine
1−Methyladenosine
5'−Methylthioadenosine
N2,N2−Dimethylguanosine
Adenosine
Modality
EE
RE
Timepoint
during 20 min
during 40 min
post 10 min
post 30 min
post 3.5 hr
post 24 hr
Z−Score
21.4
0
−1.8
B RefMet C24 Bile Acids
Modality
Timepoint
Muricholic acid
Hyocholic acid
Hyodeoxycholic acid
Ursodeoxycholic acid
Cholic acid
Taurochenodeoxycholic acid
Taurodeoxycholic acid
Glycocholic acid
Taurocholic acid
Lithocholic acid
Taurohyodeoxycholic acid
Glycodeoxycholic acid
Glycodeoxycholic acid/Glycochenodeoxycholic acid
Glycochenodeoxycholic acid
Deoxycholic acid
Tauroursodeoxycholic acid
Glycoursodeoxycholic acid
Glycolithocholic acid
Glycohyodeoxycholic acid
Tauromuricholic acid
Modality
EE
RE
Timepoint
during 20 min
during 40 min
post 10 min
post 30 min
post 3.5 hr
post 24 hr
Z−Score
2.7
0
−4.3

Figure S3

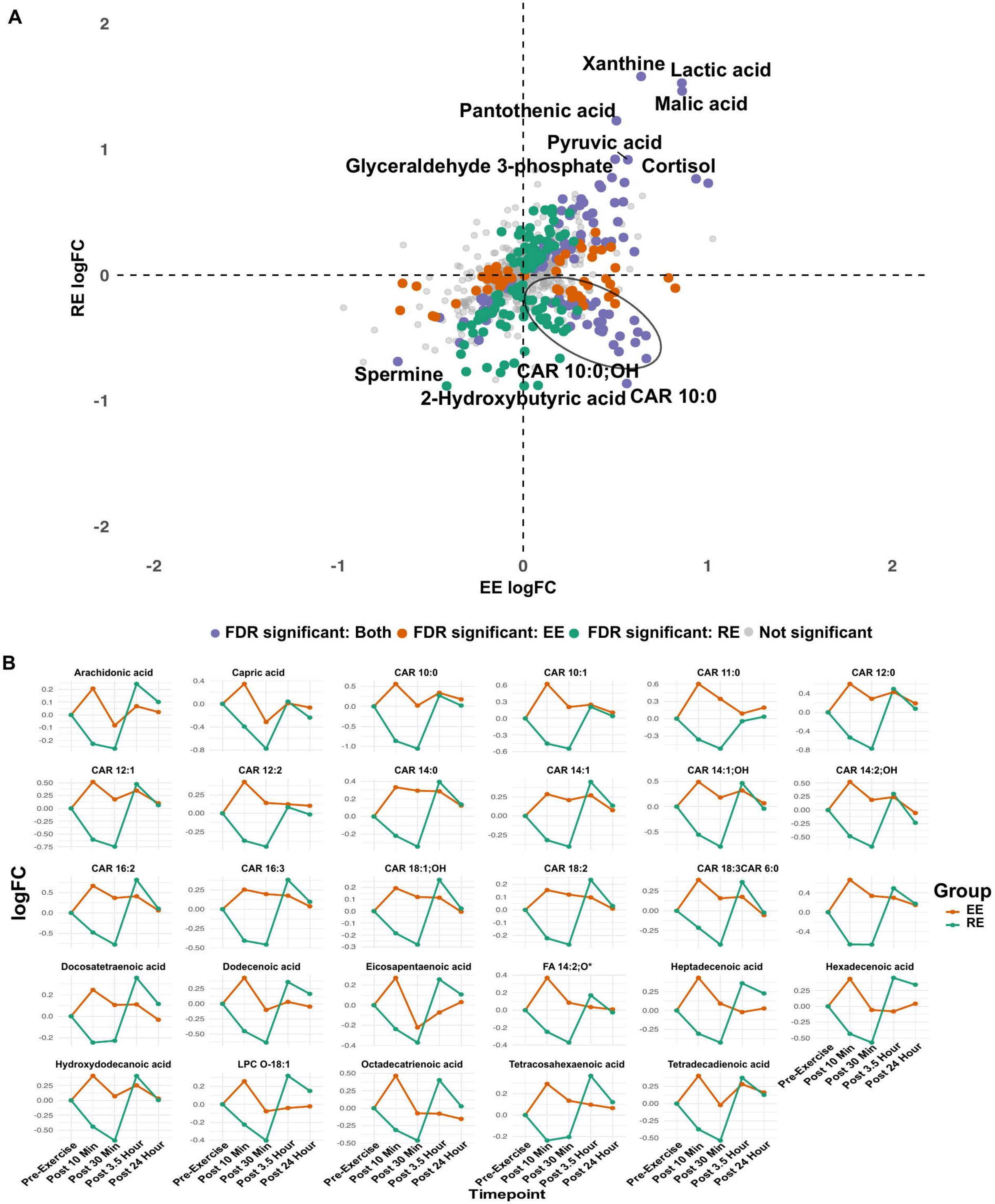

A
Xanthine
Lactic acid
Malic acid
Pantothenic acid
Pyruvic acid
Glyceraldehyde 3-phosphate
Cortisol
Spermine
CAR 10:0;OH
2-Hydroxybutyric acid
CAR 10:0
RE logFC
EE logFC
FDR significant: Both
FDR significant: EE
FDR significant: RE
Not significant
B
Arachidonic acid
Capric acid
CAR 10:0
CAR 10:1
CAR 11:0
CAR 12:0
CAR 12:1
CAR 12:2
CAR 14:0
CAR 14:1
CAR 14:1;OH
CAR 14:2;OH
CAR 16:2
CAR 16:3
CAR 18:1;OH
CAR 18:2
CAR 18:3CAR 6:0
Docosatetraenoic acid
Dodecenoic acid
Eicosapentaenoic acid
FA 14:2;O*
Heptadecenoic acid
Hexadecenoic acid
Hydroxydodecanoic acid
LPC O-18:1
Octadecatrienoic acid
Tetracosahexaenoic acid
Tetradecadienoic acid
logFC
Timepoint
Pre-Exercise
Post 10 Min
Post 30 Min
Post 3.5 Hour
Post 24 Hour
Group
EE
RE

Figure S5

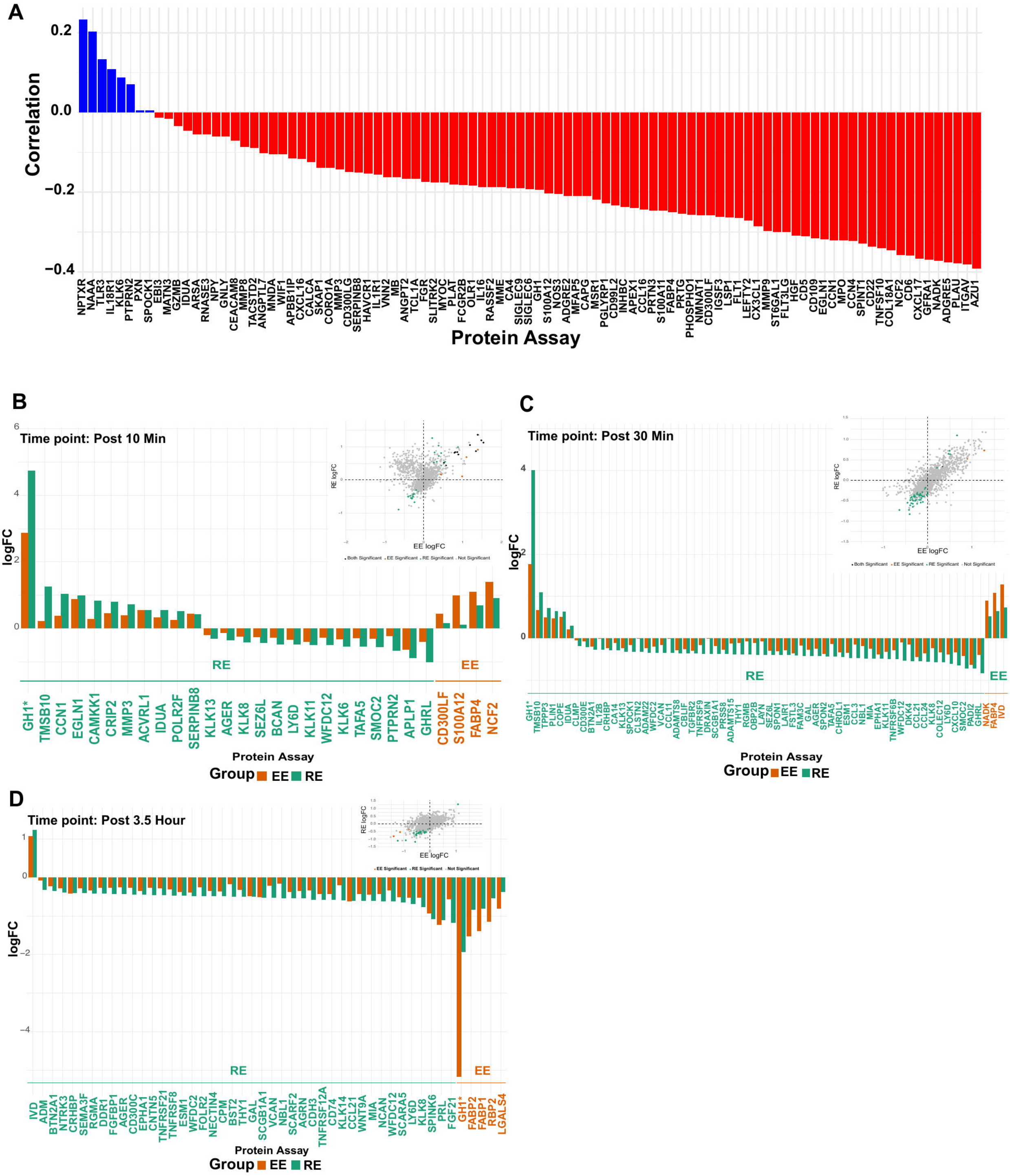
A
0.2
0.0
−0.2
−0.4
Correlation
NPTXR NAAA TLR3 IL18R1 KLK6 PTPRN2 PXN SPOCK1 EBI3 MATN3 GZMB IDUA ARSA RNASE3 NPY GNLY CEACAM8 MMP8 TACSTD2 ANGPTL7 MNDA WIF1 APBB1IP CXCL16 CALCA SKAP1 CORO1A MMP3 CD300LG SERPINB8 HAVCR1 IL1R1 VNN2 MB ANGPT2 TCL1A FGR SLITRK2 MYOC PLAT FCGR2B OLR1 IL16 RASSF2 MME CA4 SIGLEC9 SIGLEC6 GH1 S100A12 NOS3 ADGRE2 MFAP5 CAPG MSR1 PGLYRP1 CD99L2 INHBC APEX1 CCL16 PRTN3 S100A11 FABP4 PRTG PHOSPHO1 NMNAT1 CD300LF IGSF3 LSP1 FLT1 LEFTY2 CX3CL1 MMP9 ST6GAL1 FLT3LG HGF CD5 CD109 EGLN1 CCN1 MPO CCN4 SPINT1 CD22 TNFSF10 COL18A1 NCF2 CD6 CXCL17 GFRA1 NADK ADGRE5 PLAU ITGAV AZU1
Protein Assay
B
Time point: Post 10 Min
6
4
2
0
logFC
RE
EE
GH1* TMSB10 CCN1 EGLN1 CAMKK1 CRIP2 MMP3 ACVRL1 IDUA POLR2F SERPINB8 KLK13 AGER KLK8 SEZ6L BCAN LY6D KLK11 WFDC12 KLK6 TAFA5 SMOC2 PTPRN2 APLP1 GHRL CD300LF S100A12 FABP4 NCF2
Protein Assay
Group EE RE
RE logFC
EE logFC
Both Significant EE Significant RE Significant Not Significant
C
Time point: Post 30 Min
4
2
0
logFC
RE
EE
GH1* TMSB10 TPPP3 PLIN1 COPE IDUA CLMP CD300E BTN2A1 IL12B CRHBP CA14 KLK13 SPOCK1 CLSTN2 ADAM22 WFDC2 VCAN CCL11 ADAMTS8 CBLIF TGFBR2 TNFRSF9 DRAXIN SCGB1A1 PRSS8 ADAMTS15 THY1 RGMB OBP2B LAYN SEZ6L SPON1 LAIR1 FSTL3 FAM3C GAL AGER SPON2 TAFA5 CHRDL1 ESM1 CCL3 NBL1 MIA EPHA1 KLK11 TNFRSF6B WFDC12 DKK4 CCL21 CCL24 KLK8 COLEC12 LY6D CXCL10 SMOC2 PADI2 GHRL NADK FABP4 IVD
Protein Assay
Group EE RE
RE logFC
EE logFC
Both Significant EE Significant RE Significant Not Significant
D
Time point: Post 3.5 Hour
1
0
−2
−4
logFC
RE
EE
IVD ADM BTN2A1 NTRK3 CRHBP SEMA3F RGMA DDR1 FGFBP1 AGER CD300C EPHA1 CNTN5 TNFRSF21 TNFRSF8 ESM1 WFDC2 FOLR2 NECTIN4 CPM BST2 THY1 GAL SCGB1A1 VCAN NBL1 SCARF2 AGRN CDH3 TNFRSF12A CD74 KLK14 CCL21 WNT9A MIA NCAN WFDC12 SCARA5 LY6D KLK8 SPINK6 PRL FGF21 GH1* FABP2 FABP1 RBP2 LGALS4
Protein Assay
Group EE RE
RE logFC
EE logFC
EE Significant RE Significant Not Significant

Figure S6

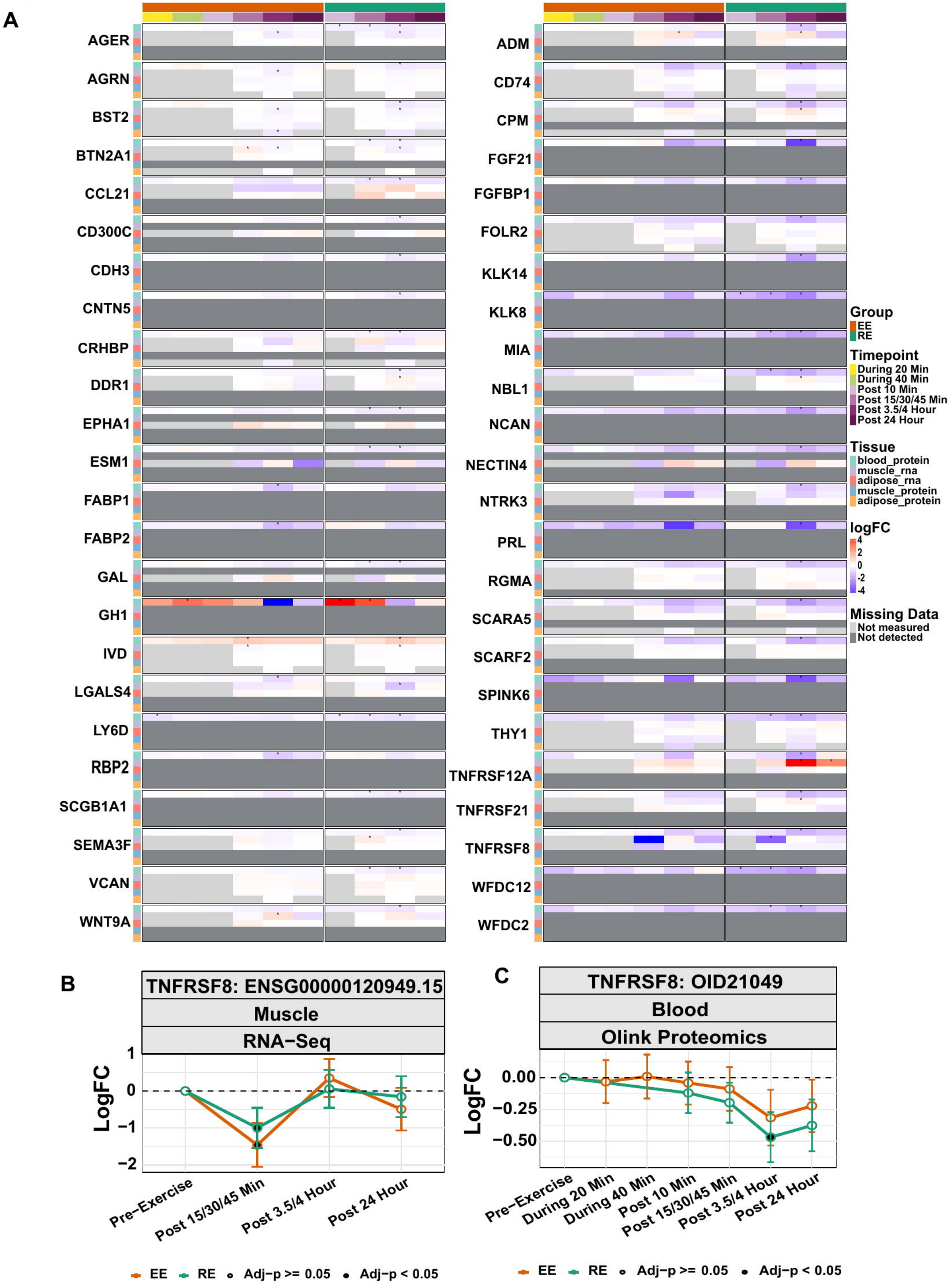
A
AGER
AGRN
BST2
BTN2A1
CCL21
CD300C
CDH3
CNTN5
CRHBP
DDR1
EPHA1
ESM1
FABP1
FABP2
GAL
GH1
IVD
LGALS4
LY6D
RBP2
SCGB1A1
SEMA3F
VCAN
WNT9A
ADM
CD74
CPM
FGF21
FGFBP1
FOLR2
KLK14
KLK8
MIA
NBL1
NCAN
NECTIN4
NTRK3
PRL
RGMA
SCARA5
SCARF2
SPINK6
THY1
TNFRSF12A
TNFRSF21
TNFRSF8
WFDC12
WFDC2
Group
EE
RE
Timepoint
During 20 Min
During 40 Min
Post 10 Min
Post 15/30/45 Min
Post 3.5/4 Hour
Post 24 Hour
Tissue
blood_protein
muscle_rna
adipose_rna
muscle_protein
adipose_protein
logFC
4
2
0
-2
-4
Missing Data
Not measured
Not detected
B
TNFRSF8: ENSG00000120949.15
Muscle
RNA−Seq
LogFC
1
0
−1
−2
Pre−Exercise
Post 15/30/45 Min
Post 3.5/4 Hour
Post 24 Hour
EE
RE
Adj−p >= 0.05
Adj−p < 0.05
C
TNFRSF8: OID21049
Blood
Olink Proteomics
LogFC
0.00
−0.25
−0.50
Pre−Exercise
During 20 Min
During 40 Min
Post 10 Min
Post 15/30/45 Min
Post 3.5/4 Hour
Post 24 Hour
EE
RE
Adj−p >= 0.05
Adj−p < 0.05

Figure S7

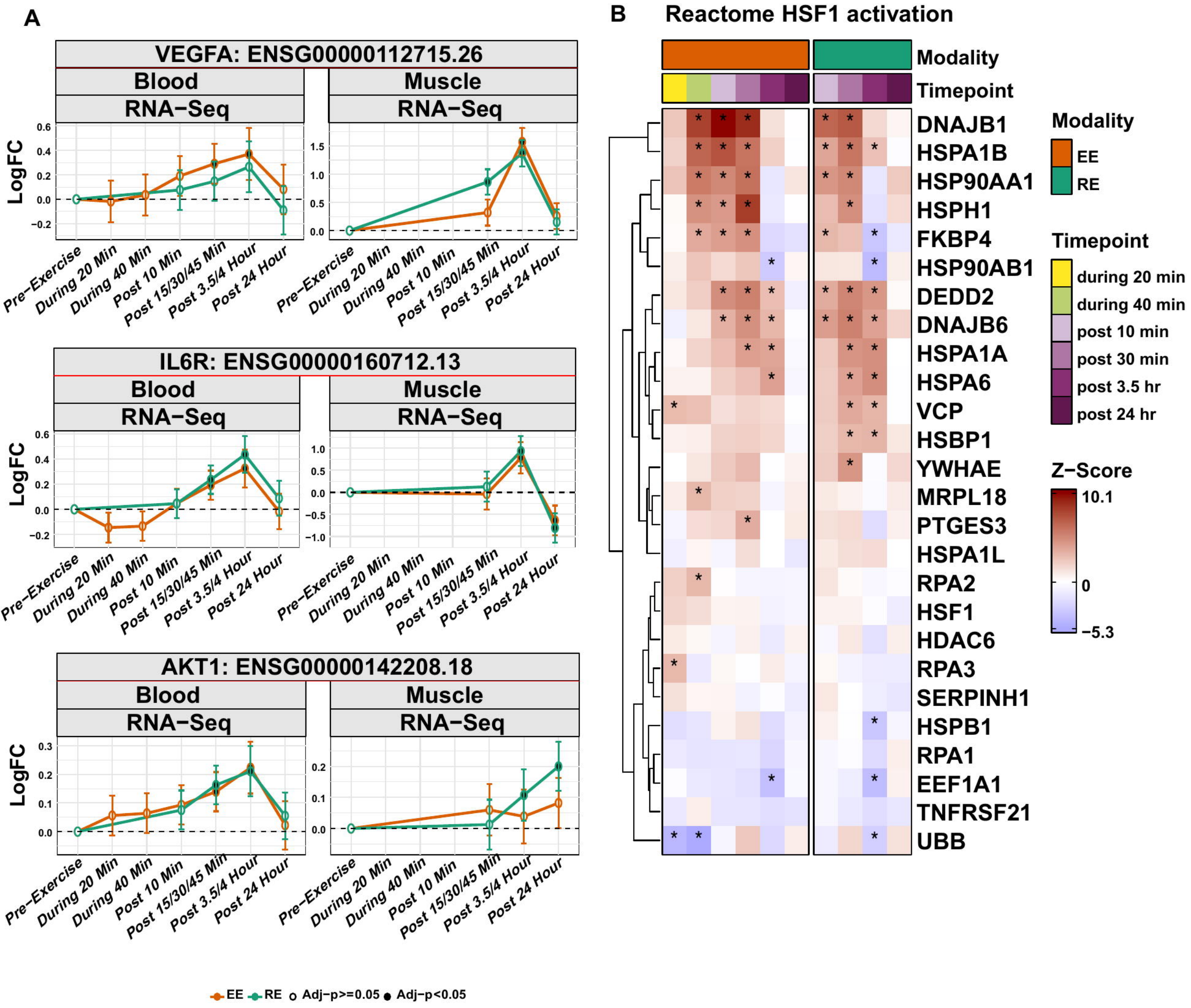
A
VEGFA: ENSG00000112715.26
IL6R: ENSG00000160712.13
AKT1: ENSG00000142208.18
Blood
Muscle
RNA−Seq
LogFC
Pre−Exercise
During 20 Min
During 40 Min
Post 10 Min
Post 15/30/45 Min
Post 3.5/4 Hour
Post 24 Hour
EE
RE
Adj−p>=0.05
Adj−p<0.05
B
Reactome HSF1 activation
Modality
Timepoint
DNAJB1
HSPA1B
HSP90AA1
HSPH1
FKBP4
HSP90AB1
DEDD2
DNAJB6
HSPA1A
HSPA6
VCP
HSBP1
YWHAE
MRPL18
PTGES3
HSPA1L
RPA2
HSF1
HDAC6
RPA3
SERPINH1
HSPB1
RPA1
EEF1A1
TNFRSF21
UBB
during 20 min
during 40 min
post 10 min
post 30 min
post 3.5 hr
post 24 hr
Z−Score
10.1
0
−5.3

Figure S8

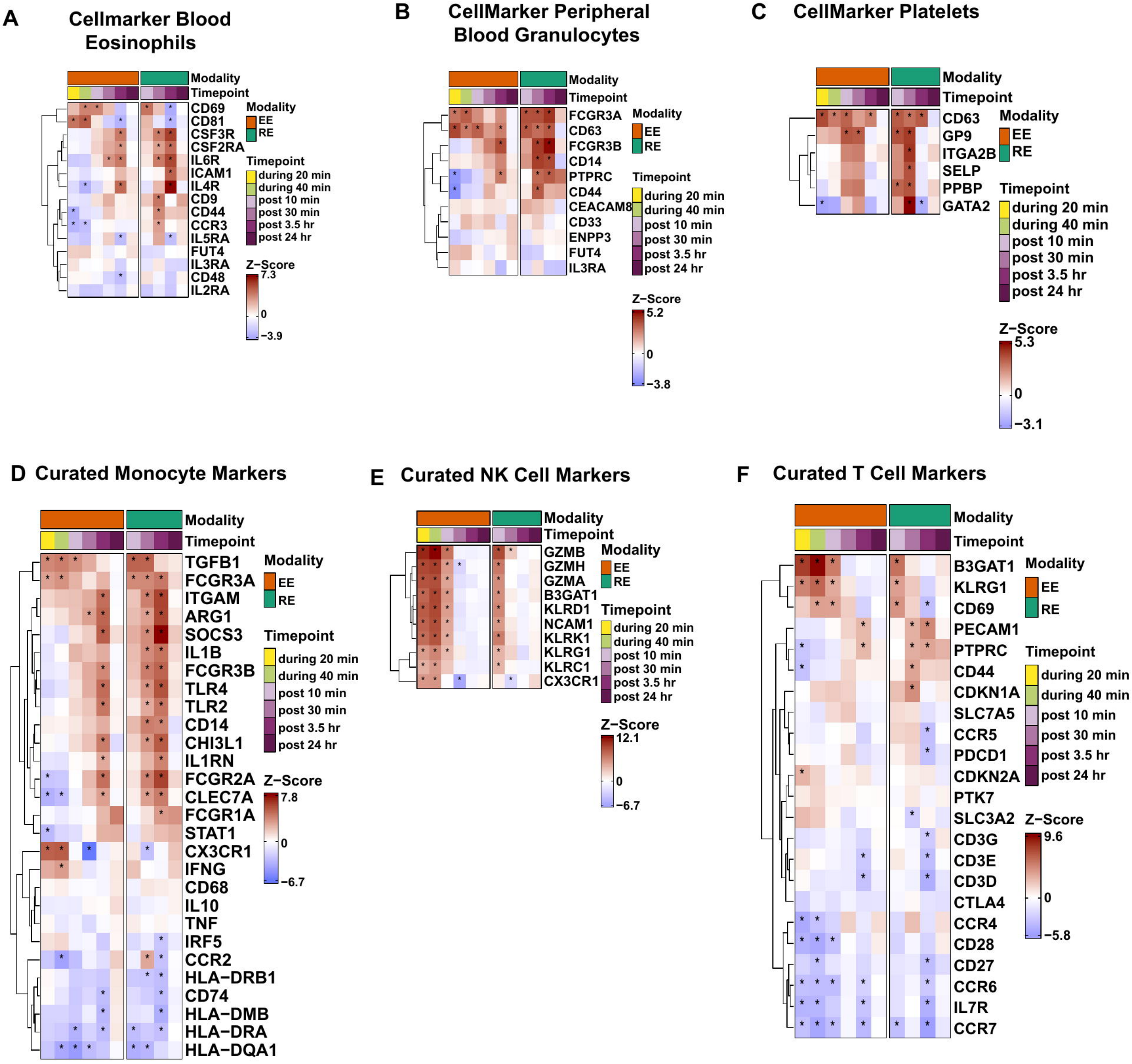
A
Cellmarker Blood Eosinophils
Modality
Timepoint
CD69
CD81
CSF3R
CSF2RA
IL6R
ICAM1
IL4R
CD9
CD44
CCR3
IL5RA
FUT4
IL3RA
CD48
IL2RA
Modality
EE
RE
Timepoint
during 20 min
during 40 min
post 10 min
post 30 min
post 3.5 hr
post 24 hr
Z-Score
7.3
0
−3.9
B
CellMarker Peripheral Blood Granulocytes
Modality
Timepoint
FCGR3A
CD63
FCGR3B
CD14
PTPRC
CD44
CEACAM8
CD33
ENPP3
FUT4
IL3RA
Modality
EE
RE
Timepoint
during 20 min
during 40 min
post 10 min
post 30 min
post 3.5 hr
post 24 hr
Z-Score
5.2
0
−3.8
C
CellMarker Platelets
Modality
Timepoint
CD63
GP9
ITGA2B
SELP
PPBP
GATA2
Modality
EE
RE
Timepoint
during 20 min
during 40 min
post 10 min
post 30 min
post 3.5 hr
post 24 hr
Z-Score
5.3
0
−3.1
D
Curated Monocyte Markers
Modality
Timepoint
TGFB1
FCGR3A
ITGAM
ARG1
SOCS3
IL1B
FCGR3B
TLR4
TLR2
CD14
CHI3L1
IL1RN
FCGR2A
CLEC7A
FCGR1A
STAT1
CX3CR1
IFNG
CD68
IL10
TNF
IRF5
CCR2
HLA−DRB1
CD74
HLA−DMB
HLA−DRA
HLA−DQA1
Modality
EE
RE
Timepoint
during 20 min
during 40 min
post 10 min
post 30 min
post 3.5 hr
post 24 hr
Z-Score
7.8
0
−6.7
E
Curated NK Cell Markers
Modality
Timepoint
GZMB
GZMH
GZMA
B3GAT1
KLRD1
NCAM1
KLRK1
KLRG1
KLRC1
CX3CR1
Modality
EE
RE
Timepoint
during 20 min
during 40 min
post 10 min
post 30 min
post 3.5 hr
post 24 hr
Z-Score
12.1
0
−6.7
F
Curated T Cell Markers
Modality
Timepoint
B3GAT1
KLRG1
CD69
PECAM1
PTPRC
CD44
CDKN1A
SLC7A5
CCR5
PDCD1
CDKN2A
PTK7
SLC3A2
CD3G
CD3E
CD3D
CTLA4
CCR4
CD28
CD27
CCR6
IL7R
CCR7
Modality
EE
RE
Timepoint
during 20 min
during 40 min
post 10 min
post 30 min
post 3.5 hr
post 24 hr
Z-Score
9.6
0
−5.8
